# An Assessment of Sociodemographic Factors, Attitude, Knowledge, and Practices of the Elderly’s Caregivers with Respect to Elderly Food Safety

**DOI:** 10.3390/foods14244212

**Published:** 2025-12-08

**Authors:** Wing-Tung Leung, Sirui Li, Peter Hoi-Fu Yu, Shun-Wan Chan

**Affiliations:** 1 Faculty of Science, The University of Hong Kong, Hong Kong, China; u3556531@connect.hku.hk (W.-T.L.); u3637606@connect.hku.hk (S.L.); 2 Department of Food and Health Sciences, Technological and Higher Education Institute of Hong Kong, Hong Kong, China; bcpyu1@gmail.com

**Keywords:** food poisoning, food safety, caregivers, elderly, knowledge, attitude, practice

## Abstract

In the past decades, outbreaks of food poisoning have been a significant concern in Hong Kong. Under the stressful workplace culture, people nowadays overlook the importance of food safety and food handling practices. Elderly people are at a higher risk of foodborne illness among the infected groups due to weakened immunity. It is important for the elderly’s caregivers to be equipped with good food safety knowledge and food handling practices in order to ensure the food safety of the elderly. An online questionnaire was distributed in Hong Kong using online platforms with the aim of investigating the sociodemographic characteristics, attitudes, knowledge, and handling practices with respect to elderly food safety among 390 participants who are the elderly’s caregivers. These factors represent different backgrounds of the elderly’s caregivers, and the questionnaire provides evidence to support how food safety knowledge and attitudes of elderly’s caregivers associate with elderly food safety. By investigating them, it helps the public understand the significance of preventing food poisoning outbreaks. Thus, the public health of the elderly can be improved. It was observed that the elderly’s caregivers with a higher level of education had better knowledge of food safety in elderly care and better food handling practices. Their attitudes had a strong positive correlation with their knowledge and handling practices towards food safety. This study emphasized that food safety knowledge and practices of the elderly’s caregivers in Hong Kong should be improved effectively by enforcing stricter regulations on elderly food safety protocols, raising public awareness on elderly food safety and implementing tailored educational programmes according to the background of the elderly’s caregivers.

## 1. Introduction

Under the stressful workplace culture, the fast-paced lifestyle has now become mainstream in Hong Kong. Some people even find it challenging to spend time cooking and eating. However, this can threaten food safety as people always prioritize speed over the proper handling of food. The risk level of foodborne illness is particularly high in the elderly, indicating the importance of ensuring elderly food safety. The outbreak of food poisoning has been a significant concern in Hong Kong. According to the Centre for Food Safety, the number of reported food poisoning outbreaks (FPO) in Hong Kong fluctuated from 117 to 256 cases per year from 2015 to 2024 [1]. With the recurring incidents of food poisoning, there is a pressing need to investigate and mitigate the FPO in Hong Kong. Foodborne illness causes gastrointestinal symptoms, such as diarrhea, vomiting, abdominal pain, nausea, and fever [2,3]. Most of the FPOs are caused by viruses and bacteria. For example, norovirus is the most common causative agent for FPO in Hong Kong in 2024, increasing about 1.8 times from 25.7% to 46.9% of all FPO between 2023 and 2024 [1]. The alarming increase in FPO urges immediate action to strengthen public health.

It is suggested that elderly people aged 65 and above are at higher risk of foodborne illness due to weakened immunity and decreased gastric acid secretion [4,5]. In *Listeria monocytogenes* infection, the mortality rate among elderly people (45.2%) is much higher than that among people aged below 65 (15.4%) [6,7]. Foodborne illness can bring life-threatening effects, especially to elderly people. Elderly food safety should not be neglected, as the population aged 65 and above is projected to increase from 1.5 million in 2021 to 2.52 million in 2039 [8]. The rapid growth in the ageing population may result in an increase in the number of elderly people suffering from foodborne diseases.

While most of the elderly people have lost their functional independence and may rely on their caregivers for daily activities, including feeding, dressing, and bathing, the food safety knowledge of the caregivers is of utmost importance to ensure the food safety of the elderly people. Caregivers of the elderly people can be formal caregivers, such as health professionals in health institutions, or informal caregivers, including family members or friends who have not received food safety training for elderly people [9]. They provide personal care and health care to elderly people, ranging from meal preparation to medications. According to the report of the Census and Statistics Department (2023), 70% of the elderly were living with their primary caregivers, such as spouses, sons, or daughters [10]. Hiring foreign domestic helpers from the Philippines, Indonesia, and Thailand through the Immigration Department is also very common in Hong Kong. These informal caregivers usually lack knowledge of food safety and handling practices as they have not received any professional food safety training. As 70% of the elderly suffer from one or more chronic disease(s) in Hong Kong, a large proportion of them have lost functional independence, which becomes a major concern for the elderly [11]. As a result, many Hong Kong families will hire a caregiver to provide a daily diet to the elderly, while following safe food selection and proper food handling practices. For example, caregivers should provide soft food to elderly people with weak swallowing or chewing ability, as they are prone to choking, which may lead to suffocation [12]. The elderly’s caregivers should pay attention to the food handling practices when preparing meals for the elderly. Nevertheless, many caregivers may not be equipped with good food safety knowledge. In total, 278 cases (20.9%) in the residential care homes for the elderly were recorded, among a total of 1328 acute gastroenteritis outbreaks caused by infections from viruses, bacteria, or parasites from 2012 to 2021 [13]. Therefore, it is crucial for the elderly’s caregivers to be well-trained with food safety knowledge and food handling practices to overcome the challenge of foodborne illness in the elderly.

Research in Turkey and Brazil has shown that the elderly’s caregiver’s food handling practices have been associated with their food safety knowledge, attitude, and education level [9]. However, there is a lack of available research on this topic in Asia, including Hong Kong. As sociodemographic factors and lifestyle are regionally dependent, the study results may vary across countries. In Hong Kong, major contributing factors in all FPO are relevant to the food handling practices, including the consumption of raw food, inadequate cooking, and contamination by utensils. This shows that most of the caregivers do not have proper food handling practices. With rapid cultural, economic, and social development, Hong Kong has been an international city with a diverse population. People have been educated with a combination of Chinese and Western cultures. Consequently, their distinct background have led to a large variation in food handling practices among the elderly’s caregivers. There is always a misconception that all food can be kept fresh forever as long as it is kept in the refrigerator. Yet, there is a time limitation for food preservation. Food that is expired or leftovers being kept for three days should be discarded immediately to avoid contamination from the spoiled food, which increases the risk of foodborne illness [14]. Thereby, it is necessary to improve the food handling practice of the elderly’s caregivers in Hong Kong.

Previous findings in Nevada’s study assessing the food safety behaviour of caregivers at elderly care facilities suggested that the elderly’s caregivers had some basic understanding of food safety [15]. Nevertheless, the study only targeted the caregivers in the elderly care facilities. The findings may not totally reflect the real situation of food safety knowledge of the elderly’s caregivers population located in that region. The targeted participants should involve all the elderly’s caregivers, including those caring for the elderly at home, to have a more comprehensive understanding of caregivers’ food safety knowledge in that region. Furthermore, the study did not gather observational data about the elderly’s caregivers. Such information would be helpful for comparing individual responses, which aided the investigation into food safety knowledge among each of the elderly’s caregivers. In view of the limitations of the previous study, it is important to conduct research in Hong Kong to assess the food safety knowledge and practices within the elderly’s caregiver population for a more comprehensive understanding of the current situation. The sociodemographic factors of the elderly’s caregivers should be included to evaluate the associations between food safety knowledge and practices of the elderly’s caregivers.

Various studies suggested that the food safety knowledge of the elderly’s caregivers may be affected by training frequency, duration of elderly caring and sociodemographic factors, including age, gender, education level, and income [9,16]. Food safety knowledge regarding foodborne disease, personal hygiene, and food handling is significant in safeguarding food safety. Food safety training given by health professionals provides food safety knowledge and food handling practices to the elderly’s caregivers. Caregivers with previous experience in elderly care also reveal professional food handling practices, which are associated with their food safety knowledge. In addition, the educational level of the caregivers may help determine their knowledge level in food safety, such as preventing foodborne disease and maintaining personal hygiene. Consequently, the elderly’s caregivers take up an important role in ensuring elderly food safety by preventing food contamination. However, studies concerning the food safety knowledge of the elderly’s caregivers in Hong Kong are limited. It is crucial for us to understand the food safety knowledge of the elderly’s caregivers through various perspectives to address the problems in elderly food safety.

Public awareness of food safety is directly guided by people’s beliefs and behaviours. Facts were published with accurate details through academic expression, such as statistics, but they were less helpful in addressing the food safety problems, due to different educational levels. So, under the sociodemographic background of Hong Kong, academic knowledge level and personal behaviours were established as two indicators in this assessment. The comparison may clarify the factors, so that appropriate improvements can be matched with the real needs.

The causal relationship of an event is multi-dimensional, rather than linear. Therefore, in terms of questionnaire design, the study is fundamentally outlined by the elderly’s caregivers’ attitudes, knowledge, and practices. Attitude reflects the public awareness of the importance of food safety, and knowledge assesses people’s command of scientific food safety knowledge. Then, in the practice section, their real behaviours were recorded to contrast attitudes and knowledge. Good attitudes could influence the willingness of people to learn from academic food safety knowledge, as well as the willingness to take on its practical advantages. However, the lack of professionals also creates barriers in recognizing proper, safe food handling, and an abundance of knowledge does not mean that people will implement the practice.

This study aims to evaluate the attitudes, knowledge, and handling practices of elderly’s caregivers with respect to food safety, by assessing sociodemographic factors, attitudes, knowledge, and practices. It is known that understanding these factors is vital for developing strategies for food safety in Hong Kong, in terms of regulations, promotion, and education. Therefore, the government can utilize the findings of this study to establish food safety guidelines regarding elderly food safety, in order to improve caregivers’ food safety knowledge and prevent FPO among the elderly people in Hong Kong.

## 2. Materials and Methods

### 2.1. Study Design

The research was conducted in a quantitative-descriptive design, based on the perception and attitudes of the elderly’s caregivers regarding food safety for the care of elderly people. A self-designed questionnaire, comprising 39 multiple-choice questions, was developed and structured into four sections: (i) participant demographics, (ii) attitudes toward elderly food safety, (iii) food safety knowledge, and (iv) food safety handling practices. The study design was informed by the Knowledge–Attitude–Practice model. It postulates that knowledge is a foundational determinant of attitudes and subsequent practices. The questionnaire was distributed via online platforms to a non-random, convenience sample in Hong Kong during June and July 2025. Participants completed the questionnaire by themselves in 5 to 10 min. An ethical approval by the Human Research Ethics Committee at the University of Hong Kong was obtained on 3 July 2025, before the study was conducted. The statistical analysis provides evidence for informing risk-based prioritization in elderly’s caregivers’ training and policy development.

### 2.2. Sample Size and Recruitment

The aim of the study was to investigate the sociodemographic factors, attitudes, knowledge, and handling practices of the elderly’s caregivers with regard to elderly food safety in a comprehensive representation. The target participants in this study were Hong Kong residents aged 18 or above, who have been living in Hong Kong for at least 6 months, and have been caring for elderly people aged 65 or above. Participants were informed in the online questionnaires about the purpose of the study, and online consent was obtained from them before proceeding with the data collection. If the participants did not fully understand the questions in the questionnaires, they were explained by the researchers. The background of the participants was surveyed to ensure they fulfilled the study requirement.

The sample size of the study should be determined cautiously, as it was directly associated with the quality of the data, affecting the accuracy and reliability of the study. The sample size was calculated using the standard error Formula (1) suggested by a previous study [17].

(1) $$n=\frac{Z^{2}\times N\times P\times (1-P)}{d^{2}\times \left(N-1\right)+{\;Z}^{2}\times P\times (1-P)}$$

According to the Census and Statistics Department, the Hong Kong population is provisionally estimated at 7,534,200 at the end of 2024 [18]. Considering a confidence interval of 95% (*Z* = 1.96), the population size (*N* = 7,534,200), the sample portion (*P* = 50%) and the degree of accuracy (*d* = 0.05), an ideal sample size was calculated as $\frac{1.96^{2}\;\times \;7,534,200\times (0.5)\times (1\;-\;0.5)}{{\left(0.05\right)}^{2}\times \left(7,534,200\;-\;1\right)\;+\;{\left(1.96\right)}^{2}\times (0.5)\times (1\;-\;0.5)}$ ≈ 384 using Equation (1) [1]. Therefore, 390 participants in total were recruited for this study.

### 2.3. Food Safety Knowledge and Handling Practice of the Respondents

To evaluate the food safety knowledge of the elderly’s caregivers, a summary score for the Food Safety Knowledge Test in Section 3 of the questionnaire was calculated. The Food Safety Knowledge Test is designed based on the Hazard Analysis and Critical Control Points (HACCP) food safety standard. It consisted of 9 multiple-choice questions based on three topics: (i) Food Temperature Control and Foodborne Diseases, (ii) Personal and Environmental Hygiene, and (iii) Food Handling. Each topic consisted of 3 questions using the ordinal scoring scale. Each correct answer earned 1 mark, while no mark was deducted for a wrong answer. In a total number of 9 questions, respondents scoring 0–3 marks were regarded as “Poor”, those gaining 4–6 marks were considered as “Fair”, and those receiving 7–9 marks were considered as “Good”. The actual scores obtained by the respondents in the Food Safety Knowledge Test were compared with the expected scores perceived by themselves. Similarly, 9 multiple-choice questions were asked to examine the food handling practices of the elderly’s caregivers based on their behaviours towards food safety.

### 2.4. Data Analysis Tools

The data collection was conducted using an online questionnaire on Google Forms comprising multiple-choice questions. The results were summarized in the form of graphs using GraphPad Prism Version 10 for Windows. All statistical analyses were performed by IBM SPSS Statistics Version 28 for Windows. Cronbach’s alpha reliability was employed to assess the reliability and internal consistency of the questionnaire at a significance level of 0.05. The calculated Cronbach’s alpha was 0.602. Although this value falls below the conventional threshold of 0.70, such a level of reliability is not uncommon for self-designed questionnaires used in exploratory research. Consequently, despite its modest internal consistency, the scale is considered a valid and useful tool for the current analysis, though results should be interpreted with this limitation in mind. Kolmogorov–Smirnov and Shapiro–Wilk normality tests were used to investigate the data normality at the 0.05 significance level. The Chi-square test was utilized to assess the association between the study variables. Spearman’s correlation and regression analysis were used to determine the interaction between study variables at the significance level of 0.05.

## 3. Results

### 3.1. Sociodemographic Characteristics of the Respondents

To identify the sociodemographic characteristics of the 390 participants in this study, a frequency analysis was conducted. The sociodemographic characteristics of the participants were collected in Section 1 of the survey (Table 1). The number of female participants was approximately two-thirds of the total participants (73.1%), while the number of male participants made up the remaining portion (26.9%).

Respondents were predominantly aged between 45 and 54 years old (26.9%) and were married (74.1%). Regarding education level, most of the respondents had completed a high school degree (47.2%), followed by those having primary education and below (23.1%), a bachelor’s degree (21.3%), a master’s degree (7.4%), and a doctorate (1.0%). This proportionality showed that a majority of the participants were educated. Most of the respondents were employed (75.9%), followed by those retired (13.6%) and self-employed (7.7%), with a minority of them being unemployed (2.8%). With respect to monthly household income, respondents earning less than HKD 10,000 were predominant (30.5%), followed by HKD 20,000–HKD 39,999 (20.8%), HKD 40,000 or above (19.7%), and HKD 10,000–HKD 19,999 (18.5%). Only a small proportion reported being unemployed or retired (10.5%). Among the respondents, major caregivers were the sons or daughters (37.2%) and domestic helpers of the elderly (36.9%). A minority of the respondents were the relative or friend (15.1%) and the partner (10.8%) of the elderly. Meanwhile, 62.8% of the respondents had a chronic disease, with cholesterol (31.0%) identified as the most common chronic disease among the 245 caregivers having a chronic disease. The sample was predominantly caregivers who have provided elderly care for more than 36 months (51.3%), followed by 3–12 months (21.0%), 13–36 months (18.7%), and less than 3 months (9.0%).

### 3.2. Sociodemographic Profile of the Elderly Care Recipients

The sociodemographic profile of the elderly care recipient are presented in Table 2. A majority of the elderly had a chronic disease (96.4%), with diabetes being the most common chronic disease (52.1%) among the 376 elderly people with a chronic disease. Most of the elderly mildly depended on their caregivers (42.8%), followed by those moderately dependent (35.9%), severely dependent (10.5%), fully independent (6.4%), and completely dependent (4.4%). Among the elderly care recipients, a large number of them sometimes had diarrhea, vomiting, or stomachache symptoms (44.6%), followed by those who seldom (37.7%), never (12.1%), and always (5.6%) showed symptoms.

### 3.3. Respondents’ Attitudes on Food Safety Knowledge for Elderly Care

Five questions were included to understand the respondents’ actual and perceived food safety knowledge for elderly care (Table 3). The respondents were asked to rate their knowledge of food safety. Most of the respondents perceived their food safety knowledge as fair (66.7%), with a minority of them declaring their food safety knowledge as good (23.6%) and poor (9.7%). More than half of the respondents perceived their food safety knowledge as above average.

**Figure 1:**
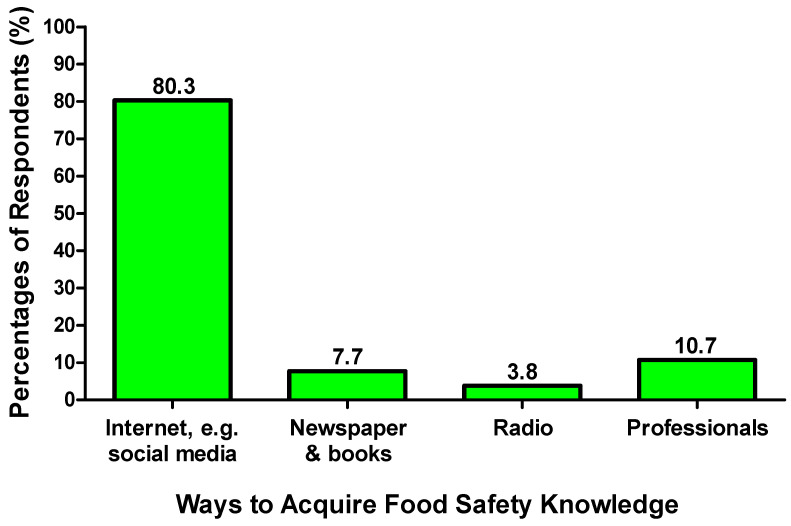
Respondents’ responses on the ways to acquire food safety knowledge.

**Figure 2:**
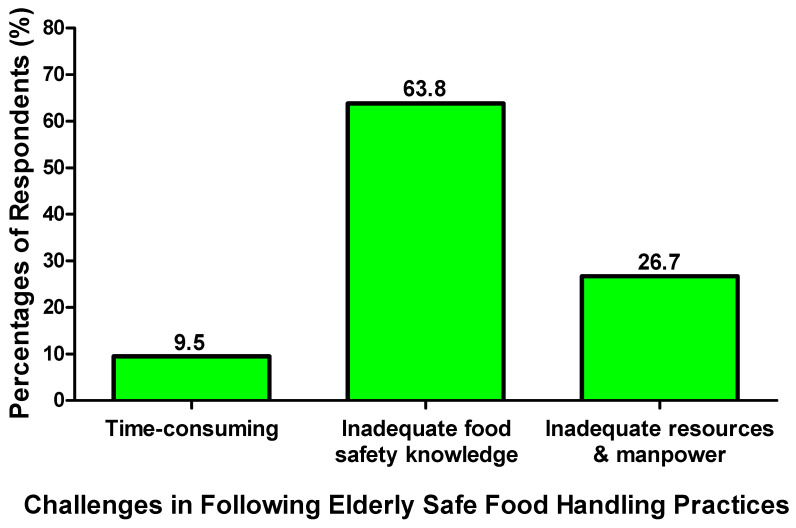
Caregivers’ biggest challenge in following safe food handling practices for elderly care.

The perceptions of the elderly’s caregivers on their food safety knowledge level might be affected by their habits of receiving information about food safety knowledge and handling practices in elderly people. A majority of the respondents sometimes obtain information about elderly food safety knowledge and handling practices (38.0%); meanwhile, 80.3% of them acquire food safety knowledge from the Internet, such as social media (Figure 1). In total, 45.9% thought that they required moderate effort in learning safe food handling practices in elderly care. In Hong Kong, maintaining safe food handling practices was a large hindrance to protecting the health of the elderly people, as it might take a lot of time to monitor food handling among the elderly’s caregivers. The elderly’s caregivers did not have sufficient food safety knowledge, while the resources and manpower of society were inadequate to implement food safety practices in elderly care. It was determined that inadequate food safety knowledge was the biggest challenge faced by the caregivers in terms of following safe food handling practices when handling food for the elderly (63.8%) (Figure 2).

### 3.4. Evaluation of Respondents’ Food Safety Knowledge for Elderly Care

With the aim of assessing respondents’ perceptions on food safety knowledge for elderly care and their true prevalence, their food safety knowledge for elderly care was evaluated by scoring their number of correct answers obtained in the Food Safety Knowledge Test. In the Food Safety Knowledge Test, nine questions were designed to assess the respondents’ food safety knowledge for elderly care (Table 4). Their food safety knowledge was assessed by their awareness on food temperature control and foodborne diseases (Question 1–3), personal and environmental hygiene (Question 4–6), as well as safe food handling (Question 7–9).

Regarding their knowledge on “Food Temperature Control and Foodborne Diseases”, it was found that more than half of the respondents lacked knowledge of the “Temperature Danger Zone” for the rapid growth of microorganisms (56.4%) and the food storage duration under room temperature (67.4%). Nearly all of them did not know the causes of foodborne diseases (96.4%). In respect of their awareness of “Personal and Environmental Hygiene” on food handling, more than half of them had a good understanding on the changing of food handling gloves (64.1%), using food-grade sanitizers in the kitchen for cleaning table surfaces (95.9%), and handling wounds during food preparation (67.7%). Concerning their understanding of “Safe Food Handling”, a majority of the respondents had a basic knowledge of providing soft or minced food to the elderly with swallowing difficulty (82.1%). Under the conditions of rapid population growth and socio-economic development, eating leftover food was common to reduce cooking time. It was believed that a significant number of respondents should acquire a basic knowledge about reheating food. However, it was surprising that most of them had a poor knowledge of the minimum temperature for reheating food (72.1%) and the concept of Hazard Analysis and Critical Control Points (HACCP) (86.4%), which is a management system that involves identifying, assessing, and controlling chemical, biological, and physical hazards to ensure food safety throughout the food chain [19].

**Figure 3:**
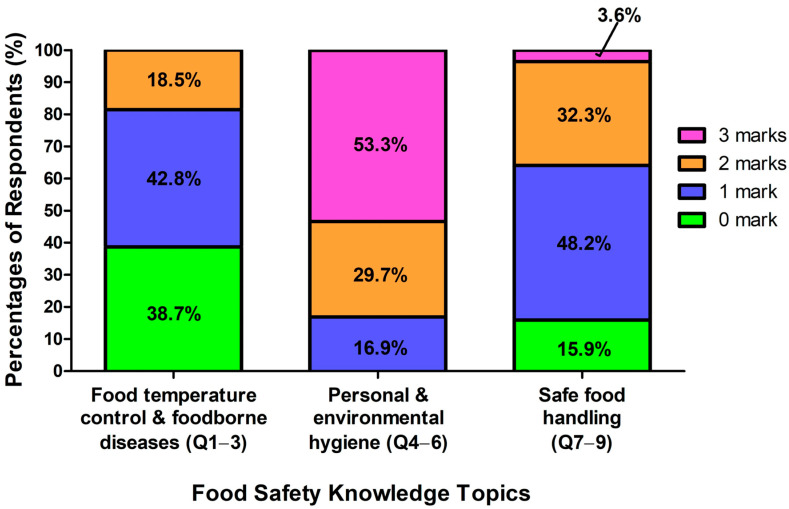
Respondents’ responses to each topic of the Food Safety Knowledge Test.

Overall, the elderly’s caregivers are most knowledgeable in “Personal and Environmental Hygiene”, with over half of the respondents (53.3%) scoring full marks in this topic (Figure 3). No one was found to have received no marks. The second knowledgeable topic is “Safe Food Handling”, with 3.6% of the respondents receiving full marks and 15.9% having no marks. The elderly’s caregivers have the least knowledge in “Food Temperature Control and Foodborne Diseases” as the proportion of respondents receiving no marks was the greatest (38.7%), compared with that of the other topics. No one attained full marks in this topic.

**Figure 4:**
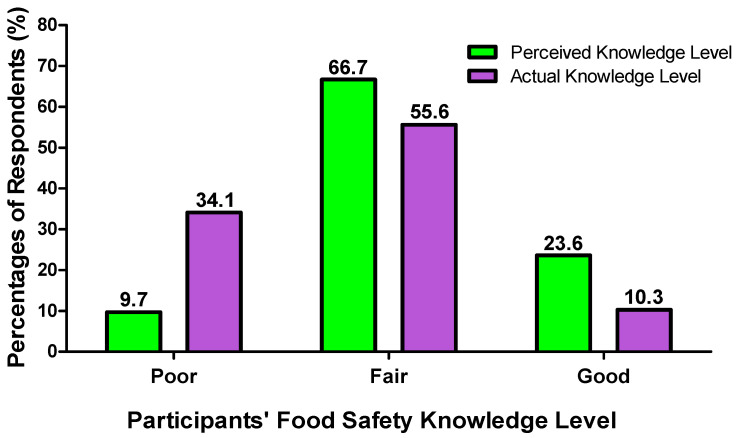
Participants’ perceived and actual food safety knowledge level.

Among the 390 respondents, those scoring “Fair” results (4–6 marks) accounted for the majority, as shown in Figure 4 (55.6%). This showed that most caregivers possessed a certain level of food safety knowledge, but still had not yet reached professional level. Their actual food safety knowledge was slightly lower than what they perceived in their food safety knowledge for elderly care, with 66.7% respondents perceiving their food safety knowledge for elderly care as fair. This showed that they had overestimated their level of food safety knowledge.

In order to evaluate the participants’ perceptions of their food safety knowledge for elderly care and their actual level of knowledge, their perceived and actual score of food safety knowledge were calculated and compared. The data showed that their actual knowledge of food safety was slightly different from the perceived knowledge of food safety reported by them. The number of participants achieving a “Good” food safety knowledge level, as determined in the Food Safety Knowledge Test (10.3%), was slightly lower than the food safety knowledge perceived by the caregivers themselves (23.6%). On the other hand, a larger number of participants obtained “Poor” food safety knowledge results (34.1%) than they perceived (9.7%). The majority of the respondents attained a “Fair” result in their actual (55.6%) and perceived (66.7%) food safety knowledge. Only the respondents who had poor food safety knowledge underestimated their knowledge.

Chi-square tests were used to determine whether the respondents’ perceived food safety knowledge is associated with their actual food safety knowledge in the population of the elderly’s caregivers (Table 5). The results showed there was a significant association between participants’ perceived and actual food safety knowledge, with a calculated chi-square (χ^2^) value (degrees of freedom = 2, *N* = 390) = 77.14 and *p*-value < 0.001). According to the Chi-Square Distribution Table, the χ^2^ critical value at a significance level of 0.001 and a degree of freedom of 2 is 13.816 [20]. Since the calculated χ^2^ value in this study was larger than the χ^2^ critical value, the null hypothesis should be rejected. The small *p*-value denoted a statistically significant result, showing strong evidence against the null hypothesis.

### 3.5. Correlations Between Sociodemographic Factors, Knowledge, and Practices

Spearman’s correlation coefficient was used to assess monotonic relationships between variables. This non-parametric test was selected for two primary reasons: first, several variables were ordinal or categorical rather than continuous, interval-level data; and second, key continuous variables, such as the practice scores, significantly deviated from a normal distribution (*p*-value < 0.001 as shown in both Kolmogorov–Smirnov and Shapiro–Wilk normality tests). A positive Spearman’s correlation coefficients shows that a variable increases when the other variable increases. The magnitude of Spearman’s correlation coefficients indicates the strength of the correlation, with a stronger association revealed in a larger Spearman’s correlation coefficient.

In this study, the correlation of 11 variables was assessed regarding sociodemographic characteristics, knowledge, and practices (Table 6). Sociodemographic factors include the elderly caregivers’ gender, age, marital status, educational level, occupation, and monthly income. Results show that “Education level” had a strong positive correlation with “Gender” (r = 0.204, *p* < 0.001), “Monthly Income” (r = 0.447, *p* < 0.001), “Food Safety Knowledge” (r = 0.614, *p* < 0.001), and “Food Safety Practice” (r = 0.294, *p* < 0.001). This indicated that participants attaining a higher educational level were mainly female, and had a higher monthly income, higher food safety knowledge, and better food safety practices. A positive relationship was also observed between “Monthly Income” and “Gender” (r = 0.221, *p* < 0.001), “Age” (r = 0.524, *p* < 0.001), and “Food Safety Knowledge” (r = 0.469, *p* < 0.001). It should be highlighted that participants who had a higher monthly income were usually female, older, and with better knowledge of food safety. This suggested that the monthly income had a strong correlation and was statistically significantly associated with the variables.

Caregiving experience included proximity to the elderly, chronic disease of caregivers, and duration of elderly caregiving. The positive correlation between “Proximity to Elderly” and “Food Safety Knowledge” (r = 0.380, *p* < 0.001) denotes that participants having a closer relationship with the elderly have better food safety knowledge. Moreover, “Duration of elderly caregiving” increased with the age of the elderly’s caregivers (r = 0.300, *p* < 0.001), indicating that the elderly’s caregivers who were older had been caring for their elderly parents for a longer period of time. It was found that the participants who were married had been caring for the elderly for a longer period of time (r = 0.275, *p* < 0.001). “Age” was strongly associated with “Chronic Disease” (r = 0.390, *p* < 0.001), suggesting that the older participants were more likely to have chronic diseases themselves.

Some sociodemographic variables demonstrated a strong relationship with the food safety knowledge. The positive relation was shown between “Food Safety Knowledge” and “Gender” (r = 0.217, *p* < 0.001) and “Food Safety Knowledge” and “Age” (r = 0.254, *p* < 0.001). This showed that good food safety knowledge was revealed in females and in older participants. According to the positive association between “Duration of elderly caregiving” and “Food Safety Knowledge” (r = 0.354, *p* < 0.001), participants who had been caring for the elderly for a longer period of time had better food safety knowledge. Similarly, they also illustrated better food safety practices as the “Duration of elderly caregiving” was positively related to “Food Safety Practice” (r = 0.246, *p* < 0.001). It was noticed that there was a strong positive association between “Food Safety Knowledge” and “Food Safety Practice” (r = 0.357, *p* < 0.001).

Food safety knowledge and practices were closely associated with sociodemographic factors and caregiving experience of the elderly’s caregivers. Sociodemographic factors showed that the elderly’s caregivers who were female, attained a higher educational level, and had a higher monthly income demonstrated good food safety knowledge and practices. However, it was also shown that caregiving experience in the elderly’s caregivers was more strongly linked to food safety knowledge and practices due to the closer proximity to the elderly and longer duration of elderly caregiving.

### 3.6. Correlations Between Attitudes, Knowledge and Practices

Respondents’ attitudes were strongly positively associated with knowledge and practices (Table 7). There was a strong correlation in “Learning Frequency” with “Perceived Knowledge” (r = 0.180, *p* < 0.001), “Food Safety Knowledge” (r = 0.244, *p* < 0.001), and “Food Safety Practice” (r = 0.115, *p* < 0.001). It indicated that participants who obtained information about food safety knowledge and handling practices more frequently perceived themselves as having better knowledge of food safety and had better food safety knowledge and food handling practices. In addition, “Dependency Level of the Elderly” was strongly correlated to “Effort Required” (r = 0.299, *p* < 0.001). It was found that the more the elderly depended on their caregivers, the more effort the caregivers were required to spend on learning safe food handling practices for elderly care. “Perceived Knowledge” was closely related to “Food Safety Knowledge” (r = 0.464, *p* < 0.001) and to “Food Safety Practice” (r = 0.204, *p* < 0.001).

However, a negative relationship was shown in “Frequency of Symptoms” with “Food Safety Knowledge” (r = −0.128, *p* < 0.001) and with “Food Safety Practice” (r = −0.122, *p* < 0.001). It was found that the elderly’s caregivers had poorer food safety knowledge and food handling practice when the elderly they cared for had frequent food poisoning symptoms, including diarrhea, vomiting, or stomachache. The strong negative relationship between “Effort Required” and “Perceived Knowledge” (r = −0.344, *p* < 0.001) and “Effort Required” and “Food Safety Knowledge” (r = −0.127, *p* < 0.001) stated that participants having poorer perceived and actual food safety knowledge thought they should spend more effort on learning safe food handling practices for elderly care.

### 3.7. Food Handling Practices and Awareness of Elderly Food Safety

**Figure 5:**
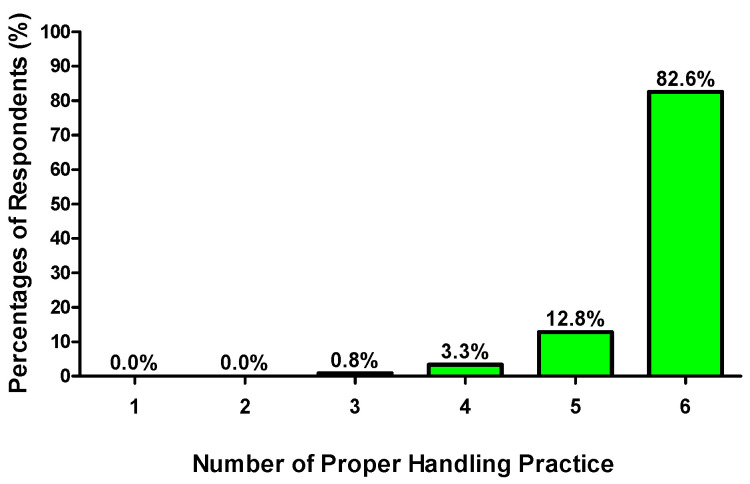
Number of proper food handling practices for elderly care performed by participants.

With respect to the food handling practices in elderly care food safety, most of the respondents revealed proper food handling practices across the five questions (Table 8). Nearly all of them would check the expiry date when purchasing and handling food (97.4%), store the raw food and cooked food separately (98.7%), wash their hands thoroughly before handling food (97.4%), wash the cooking utensils thoroughly before handling food (88.9%), and check that the food was safe before serving to the elderly (93.1%). They had good food handling practices in daily life, especially in the separation of raw food and cooked food, which revealed the highest percentage among the proper food handling practices. Only a few of the respondents did not follow appropriate food handling practices, particularly in ensuring the cleanliness of cooking utensils before handling food. Among the 390 respondents, 82.6% revealed proper food handling practices across the 5 questions on Food Safety Practice (Figure 5). Almost all respondents showed at least three proper practices in safe food handling.

In addition, the elderly’s caregivers showed different habits in elderly food handling (Table 9). A majority of the respondents reported that they would store the leftovers in the refrigerator (85.1%), while a few of them (0.5%) would not. In total, 14.4% of the elderly’s caregivers would not keep the leftovers. In maintaining environmental hygiene, more than half of them would clean and disinfect the kitchen weekly (63.5%), followed by those cleaning monthly (22.1%), daily (7.7%), and never (6.7%). Regarding caregiving to the elderly with difficulties swallowing, 17.9% of the respondents would prepare soft food or minced food for the elderly, while 10% of them would not. Most of them did not have to take care of an elderly person with difficulties swallowing (72.1%). Nearly all the respondents did not know the HACCP (91%), with only 7.5% having and 1.5% not having applied the HACCP when handling food for the elderly.

## 4. Discussion

In the present materialistic society, there is a wide variety of food choices. Yet, both the quality and quantity of food are of utmost importance. Food safety is crucial for socioeconomic development by preventing foodborne illnesses [21,22]. Foodborne disease outbreaks threaten public health, which harms the health care system and causes substantial economy losses [23]. The elderly tend to have weaker immunity and they are more susceptible to diseases [24]. Ensuring food safety is an important approach to protecting the health of the elderly population. The caregivers of the elderly should be equipped with good food safety knowledge and perform safe food handling practices in order to ensure food safety in elderly care. This current study aimed to investigate the food safety knowledge and food handling practices of the elderly’s caregivers among the population of Hong Kong, by assessing their sociodemographic factors, as well as their attitudes, knowledge, and practices of food safety.

The sociodemographic data suggested that women predominated the caregiver population (Table 1). In traditional societies, women were considered the major caregivers in the family. The gender distribution of the elderly’s caregivers was comparable to the statistics in the 2021 Population Census Thematic Report: Older Persons released by the Census and Statistics Department [10], with a distribution of 65% female and 35% male among the elderly’s caregivers. Research conducted across different countries found that women predominated among the caregivers of the elderly, accounting for 57% to 81% of all the elderly’s caregivers [25,26]. This showed that women have been considered as being centred in the home; thus, they should have a greater sense of family obligation than men. Gender was also positively associated with educational level, monthly income, and food safety knowledge (Table 6). At present, the female students enrolling in higher education programmes outnumber the male students in Hong Kong [27]. This showed that the females attaining a higher educational level might contribute to higher monthly income and equipped them with better food safety knowledge due to sufficient resources. A similar study conducted in Bangladesh also found that female food handlers demonstrated better food safety knowledge compared to male food handlers [28]. The difference in cultural norms and adherence between males and females has led to the diversity in their food safety knowledge levels. Educational level was one of the factors contributing to food safety in elderly care, according to its positive correlation with “Food Safety Knowledge” and “Food Safety Practice”. With a higher educational level, caregivers tend to have better food safety knowledge and comply with the protocols on food safety in elderly care.

Building on this educational level, the study also pointed out that the elderly’s caregivers who were older were more likely to have chronic diseases and had been taking care of the elderly for a longer period of time, demonstrating better food safety practices. Most of the elderly’s caregivers were middle-aged, ranging from 45 to 54 years old. The data was comparable with the average age of the elderly’s caregivers in Hong Kong of 54.5 years old [26]. This could be attributed to the fact that middle-aged adults were often considered to be responsible for caring for the older adults in the family due to the increasing disability of the older adults [29]. Chronic diseases were more prevalent in the elderly’s caregivers who were older [30]. The elderly’s caregivers who have chronic diseases may have more personal health concerns and have more expertise in caregiving for the elderly, who are more likely to have chronic diseases. The elderly’s caregivers who are older had a longer exposure to food safety information and more experience in food handling, thus they had a greater awareness of food safety and better adherence to safe food handling practices [31,32,33,34]. Studies suggested that younger caregivers demonstrated a lower awareness of cross-contamination prevention [31]. The accumulated experience has improved the food safety knowledge of the elderly’s caregivers, indicating that food safety knowledge level increases with age and experience of caregiving. The previous study was aligned with the findings in this current research, in that food safety knowledge improved alongside increases in the respondents’ ages and duration of elderly caregiving.

Beyond the respondents’ age, the knowledge of food safety in elderly care was shaped by the food safety attitudes and practices of the caregivers. In Hong Kong, food safety is mainly managed by the Centre for Food Safety, Food, and Environmental Hygiene Department. They have been promoting food safety knowledge and practices to enhance public awareness on food safety, implementing food surveillance programmes to identify potential hazards in food samples and protecting public health by enhancing food safety regulations [35]. It is the responsibility of the elderly’s caregivers to ensure the food is safe before serving it to the elderly. Their attitudes towards food safety determine their knowledge level of food safety. Previous research agreed with the present study in that there was a significant correlation between the knowledge level of food safety and attitudes of food handlers, but contrary to the present study, in that no correlation was found between food safety knowledge and food handling practices [36]. As only 72 food handlers participated in the previous study, the small number of participants might not be representative enough to reflect the actual situation of food safety knowledge and practices among all food handlers, compared to the current study.

A similar study conducted in the United States suggested that older adults demonstrated poor food safety behaviours, though they considered themselves knowledgeable about food safety [37]. The findings showed that older adults’ food safety behaviours did not positively associate with their food safety knowledge, which contradicted the current study. However, this could be explained by the fact that food safety knowledge of the older adults was only assessed by self-reporting. This might lead to discrepancies between the respondents’ self-reported and actual food safety knowledge, and there was no evidence showing their association. The current study evaluated both the respondents’ both perceived and actual food safety knowledge and found that they were positively correlated with their food safety practices. The caregivers had the sense that they should spend more effort on learning safe food handling practices for elderly care when the elderly they cared for depended more on them, and when they had lower perceived and actual food safety knowledge. When better food safety knowledge and food handling practices were demonstrated by the elderly’s caregivers, the elderly people they cared for exhibited a lower frequency of food poisoning symptoms.

Moreover, the elderly’s caregivers who frequently learned about food safety knowledge via the Internet, newspaper, books, radio, or professionals were found to achieve a higher food safety knowledge level and better food handling practices (Table 7). In a study investigating the effectiveness of food safety training on the behaviours of food handlers, it was found that adequate training helped to reduce improper food handling practices and mitigated the effect of food contamination on socioeconomic aspects [38,39]. This proved that learning is effective for improving food safety knowledge. Meanwhile, the higher the learning frequency was, the higher the knowledge level of food safety they acquired. In other words, they were more confident in demonstrating their food safety knowledge, explaining that they perceived a higher knowledge level in this area. These results agreed with the present study, in that respondents who perceived a higher knowledge of food safety demonstrated better food safety knowledge and handling practices.

An overall strong foundation in food safety knowledge was demonstrated by the respondents of this study. According to the results in Table 4, many handlers did not have a good perceived knowledge of “Food Temperature Control and Foodborne Diseases” in terms of food safety. They demonstrated the worst results in this topic among the three topics, with no one obtaining full marks in this topic. More than half of the respondents failed to define the “Temperature Danger Zone” for the rapid growth of microorganisms and the duration for which food should be stored under room temperature (around 25 °C). Surprisingly, only a few of them could identify the causes of foodborne diseases correctly. These results were in line with the findings in Indonesia, which indicated that research participants had poor knowledge of foodborne diseases [36]. While they believed they knew basic concepts in food temperature control, they may have overlooked the concrete knowledge on foodborne diseases, specifically the factual information. Room temperature is a key factor for foodborne diseases, since pathogenic bacteria will multiply rapidly in warm environments, especially between 40 °C and 45 °C [40,41]. Controlling the temperature appropriately can control the microbial growth in food [42]. Improper control of food temperature increases microbial hazards, causing a higher chance of foodborne diseases.

Meanwhile, “Personal and Environmental Hygiene” is the part in which the participants performed the best in the Food Safety Knowledge Test, with 53.3% attaining full marks. Almost all respondents (95.9%) knew that food-grade sanitizers are used in the kitchen to kill bacteria on the table surface. A majority of them had an understanding of changing food handling gloves after handling garbage and of covering a wound with waterproof plasters and wearing gloves when they have a wound on their hands. A study in Japan reported that most food handlers revealed a great awareness of hygiene control in food preparation, which is essential for preventing foodborne diseases [40]. A good knowledge of personal hygiene was shown among the research participants in a study from Malaysia [43]. The present study’s results with regard to hygiene knowledge are comparable to previous studies. As an international city in Asia, Hong Kong has a similar culture and environment compared with Japan and Malaysia. As a result, similar knowledge and behaviours in food hygiene were demonstrated.

In terms of “Safe Food Handling”, a relative level of knowledge was shown by the respondents. Most of the elderly’s caregivers realized that the elderly with difficulties swallowing should be provided with soft or minced food, since some of them had experience in caring for such elderly persons. However, poor knowledge was demonstrated by the respondents regarding the minimum temperature required when reheating food and the definition of the Hazard Analysis and Critical Control Points (HACCP). Food handlers might not have the practice of using food thermometers to measure the internal temperature of food [40]. It is important to ensure that the core temperature of food reaches 75 °C for reheated food, especially when serving food to vulnerable elderly people. According to Taiwan’s study, food handlers had better knowledge of food poisoning than in HACCP practice [42]. Handlers were unfamiliar with technical terms with regard to safe food handling practices [40]. Many of the elderly’s caregivers in this study did not even know about the technical term “HACCP” (91.0%). Therefore, they might fail to comprehend the question clearly, resulting in poorer results in these questions. This showed that most of the elderly’s caregivers did not have in-depth knowledge of safe food handling, particularly in the management system of food safety.

In food safety, there is still a discrepancy between learning and application. In daily life, behaviour tends to override awareness. Even though it is safer to wash one’s hands for more than 20 s each time before handling food, few participants followed the instructions, and some of them did not even clean their hands. The habit becomes persistent as people keep it long-standing. In addition to the absence of supervision, the knowledge would have failed to translate into consistent habits. Secondly, from a cognitive angle, FPOs were regarded as unlikely to happen under an individual caregiver’s care. From the questionnaire, there was a portion of participants who never cleaned the kitchen. Sometimes, when they felt stress from life, food safety was easily ignored because of emotional management. Furthermore, in theory is different from real practice. For example, it is well-known that the mixture of raw food and cooked food causes cross-contamination, but there are few details given on how to separate them properly. To extend the cultural distinction, the Chinese are used to picking vegetables themselves, which conflicts with the food safety standards. At this point, tradition is prioritized over the standard. Under the urban food environment in Hong Kong, the reliance on processed and ready-to-eat food has a great impact on caregivers’ food handling practices. Caregivers may highly depend on food delivery services due to convenience, neglecting the food safety conditions under which the food had already been prepared and stored for an extended period of time. A study conducted in Seoul suggested that a greater awareness of food hygiene was shown in citizens living in densely populated urban areas, since they had been exposed to regulatory oversight and public health campaigns [31]. This complemented the findings in the present study, in that most of the elderly’s caregivers in Hong Kong demonstrated proper food handling practices; nearly all the respondents demonstrated safe food handling practices and awareness in terms of food safety in elderly care. Respondents manifested the greatest awareness of food hygiene, as 98.7% of them would store raw food and cooked food separately to prevent cross-contamination, and would store leftovers properly in the refrigerator. A high awareness was also shown in personal and environment hygiene, owing to their behaviours in washing their hands and cooking utensils thoroughly before handling food, as well as disinfecting the kitchen on a weekly basis. Food safety conscientiousness is important to ensure food safety in elderly care, which was demonstrated in most of the participants responding that they check the expiry date when purchasing and handling food, as well as ensure that the food is safe before serving it to the elderly. Food safety awareness and safe food handling practices are especially crucial for ensuring food safety in elderly care.

In order to enhance the management system of food safety in elderly care, the government should enforce the “HACCP” system to identify potential hazards and establish critical control points when handling food for the elderly. The “HACCP” system is currently being enforced through food handler regulations by the Centre for Food Safety under the Food and Environmental Hygiene Department. Its scope should be extended to food handling in elderly care to prevent foodborne illness in the elderly and improve their health. In knowing the importance of food safety, not only will the government improve the food regulations with a stronger sense of responsibility, but it will also guarantee the quality of the food production process [44]. The enforcement by the government is key to successfully preventing improper food handling practices and improving food safety in elderly care. With strengthened food safety regulations, the food production quality will be improved [45,46]. Thus, the Hong Kong government should reinforce food safety regulations, especially in elderly care, to enhance the food safety knowledge of the elderly’s caregivers and the quality of the food served to the elderly.

In terms of public awareness, further promotion and advertising campaigns can be launched to increase public awareness of food safety in elderly care. As an authority responsible for ensuring food safety in Hong Kong, the Centre for Food Safety should run more campaigns to promote the importance of food safety in elderly care by deepening their understanding of food safety knowledge and food handling practices, as well as ways to prevent foodborne illness. They should also conduct inspections and surveillance of food premises serving the elderly. Though caregivers of the elderly may come from different backgrounds, the food safety knowledge and food handling practices they learned should be more or less the same. Researchers from China and Japan emphasized that, although cultural dietary habits vary across countries, the core principles of food safety are universal, thereby region-specific education is required to address the problem of cultural diversity [47,48,49]. As a result, all the elderly’s caregivers would be receiving the same food safety information from the Centre for Food Safety and would follow the same food safety handling practices.

Food handler training is another effective strategy to improve food safety, especially in domestic helpers caring for the elderly, since they comprise a large proportion (36.9%) of the elderly’s caregivers. Foreign domestic helpers in Hong Kong mainly come from the Philippines, Indonesia, and Thailand, and are employed through the Immigration Department. Domestic workers generally had an inadequate knowledge of food safety [50]. The Social Welfare Department, Pilot Scheme on Training for Foreign Domestic Helpers in Elderly Care, was implemented in Hong Kong in 2018 to strengthen the caregiving skills of the domestic helpers while improving the elderly’s quality of life [51]. The training consists of several modules, including food hygiene and healthy meal preparation for elderly people. As the training is free of charge, foreign domestic helpers caring for the elderly are strongly encouraged to participate in order to enhance their understanding of food safety knowledge and practices in elderly care. Some third-party companies also provide training courses regarding food safety and hygiene, including HACCP concepts to minimize risks in food preparation. As inadequate food safety knowledge is the biggest challenge for the elderly’s caregivers, it should be overcome by educating the public. Therefore, the elderly’s caregivers are encouraged to join training to enhance their food safety knowledge and handling practices.

In addition, the food safety knowledge acquired from training can help improve food handling attitudes and practices to ensure food safety. Previous studies have suggested that food safety training was significantly associated with food handlers’ attitudes in terms of food safety [36,52]. Conducting training programmes emphasizing participatory and interactive learning methods was observed to be more effective than lecture-based approaches [53]. Age-specific or culturally tailored training programme materials remarkably boosted compliance rates and knowledge retention by addressing the specific needs of the demographic [31,53]. For example, since older people may have restricted access to smartphones, they may be better educated by joining face-to-face training classes instead of online platforms. Short videos and animations can also be provided to the elderly’s caregivers. In this study, it was found that caregiving experience was more strongly linked to food safety knowledge and practices than sociodemographic factors of the elderly’s caregivers. Training programmes may be conducted in elderly homes to provide hands-on experience to the elderly’s caregivers. This further enriches their caregiving experience and improves their food safety knowledge and practices. In view of the ageing population in Hong Kong, targeted interventions can enhance public health planning and prevent foodborne illness in the elderly. Therefore, targeted and tailored food safety educational programmes are recommended for the elderly’s caregivers in a more interactive way. However, it was found that increasing the training time would significantly decrease the trained food handlers’ knowledge and practices, as this might lead to boredom, repetitiveness, and redundancy [38]. For optimal training effectiveness, the training duration should be no more than two consecutive weeks [38]. There is no guarantee that prolonged training will enhance the food safety knowledge and practices of the caregivers. It is of utmost important to evaluate the training duration while considering the effectiveness of improving food safety knowledge and practices of the elderly’s caregivers. By doing so, we hope that the food safety knowledge gap in the elderly’s caregivers across all age ranges can be narrowed.

There are several limitations in this study. First of all, the elderly’s caregivers were not asked about their literacy skills. Since some of the respondents had only received an educational level of primary or below, they might have had limited literacy skills and might not have had the ability to interpret the questionnaires clearly, even with our assistance, especially when encountering technical terms. As a result, they may have made random selections when answering the questionnaires. In future research, the literacy rate of the respondents should be considered to truly reflect the food safety knowledge and practices of the elderly’s caregivers. Secondly, discrepancies might have arisen because respondents could have interpreted the questions differently, leading to gaps between their self-reported and actual food safety knowledge and practices. This may have caused response bias and may have led to an underestimation or overestimation of the actual food safety knowledge and food handling practices. This issue is reflected in the low calculated Cronbach’s alpha value of 0.602. Enhancing the questions in the questionnaire could improve its reliability and internal consistency. Observational studies should be carried out in the future to collect more objective data by observing the groups of targeted participants without any interference, thus improving the accuracy of the self-reported knowledge and practices. Thirdly, the sample size of the current study was too small to be representative of the population. While the research was conducted in Hong Kong via social media platforms, the restricted access of social media to the older generation might have limited the generalizability and resulted in sampling bias. The research findings might not have completely reflected the population and thus lowered the result’s reliability. To mitigate this, future studies should recruit a larger number of participants in a broader geographic coverage and age groups via face-to-face interviews.

## 5. Conclusions

The current study highlights the importance of food safety knowledge and handling practices among the elderly’s caregivers, as well as their correlation with sociodemographic factors and attitudes. Female respondents, respondents with a longer duration in elderly caregiving, and respondents with positive attitudes towards food safety in elderly care generally demonstrated higher food safety knowledge and better food handling practices. In terms of food safety knowledge, more efforts should be made towards increasing knowledge and awareness about food temperature control and foodborne illness. To tackle the problem, food safety knowledge and handling practices in the elderly’s caregivers should be enhanced by enforcing the HACCP system, launching public awareness campaigns, and providing food safety training to the elderly’s caregivers. The elderly’s caregivers would be more aware of the food safety in elderly care, thus enhancing their food safety knowledge and practices, effectively improving elderly health.

The investigation into the food safety knowledge and practices of the elderly’s caregivers provides a foundation for the development of strategies, in terms of regulations, promotion, and education. While regulations reinforce food handling practices, promotion and education covering food temperature control, food hygiene, and food handling practices, as discussed in this study, help raise public awareness in food safety in elderly care and strengthen food safety knowledge among the elderly’s caregivers. It is everyone’s responsibility to ensure that the food served to the elderly is safe. The Hong Kong government should play a key role in preventing food poisoning outbreaks in the elderly by improving their caregivers’ food safety knowledge and practices. The study’s finding will inspire further research for a more comprehensive understanding in the population of the elderly’s caregivers in Hong Kong and other cities. As the sampling size was limited, more research is needed, specifically accounting for the cultural differences among the elderly’s caregivers and mitigating sampling bias in using social media platforms. Future studies considering the literacy rate of the respondents and incorporating more observational data are necessary for interpreting the results more objectively.

## Figures and Tables

**Table 1:** Sociodemographic characteristics of the participants.

Variables	Participants	Frequency, *n* (%)
Gender	Male	105 (26.9)
	Female	285 (73.1)
Age group	Below 18	0 (0)
	18–24	13 (3.3)
	25–34	78 (20.0)
	35–44	67 (17.2)
	45–54	105 (26.9)
	55–64	89 (22.8)
	64 and above	38 (9.8)
Marital status	Single	101 (25.9)
	Married	289 (74.1)
Educational level	Primary and below	90 (23.1)
	High school degree	184 (47.2)
	Bachelor’s degree	83 (21.3)
	Master’s degree	29 (7.4)
	Doctorate	4 (1.0)
Occupation	Self-employed	30 (7.7)
	Employed	296 (75.9)
	Unemployed	11 (2.8)
	Retired	53 (13.6)
Monthly household income	Less than HKD 10,000	119 (30.5)
	HKD 10,000–19,999	72 (18.5)
	HKD 20,000–39,999	81 (20.8)
	HKD 40,000 or above	77 (19.7)
	Unemployed or retired	41 (10.5)
Relationship with the elderly	Partner	42 (10.8)
	Son or Daughter	145 (37.2)
	Relative or friend	59 (15.1)
	Domestic helper	144 (36.9)
Caregivers having a chronic disease	Yes	245 (62.8)
	No	145 (37.2)
Chronic disease of caregivers	Allergy	4 (2.0)
	Coronary heart disease	15 (6.0)
	Cholesterol	76 (31.0)
	Diabetes	69 (28.0)
	Hypertension	65 (27.0)
Duration of caregiving	Less than 3 months	35 (9.0)
	3–12 months	82 (21.0)
	13–36 months	73 (18.7)
	more than 36 months	200 (51.3)

**Table 2:** Sociodemographic characteristics of the elderly care recipients.

Variables	Participants	Frequency, *n* (%)
The elderly having chronic disease	Yes	376 (96.4)
	No	14 (3.6)
Chronic disease of the elderly	Allergy	3 (0.8)
	Alzheimer	92 (24.4)
	Coronary heart disease	194 (51.6)
	Cholesterol	128 (34.0)
	Diabetes	196 (52.1)
	Hypertension	161 (42.8)
	Osteoporosis	11 (2.9)
	Cancer	1 (0.3)
	Stroke	1 (0.3)
Dependency of the elderly	Fully independent	25 (6.4)
	Mildly dependent	167 (42.8)
	Moderately dependent	140 (35.9)
	Severe dependent	41 (10.5)
	Completely dependent	17 (4.4)
Frequency of the elderly having diarrhea, vomiting or stomachache symptoms	Never	47 (12.1)
	Seldom	147 (37.7)
	Sometimes	174 (44.6)
	Always	22 (5.6)
The elderly with chronic disease	Yes	376 (96.4)
	No	14 (3.6)

**Table 3:** Attitudes toward the food handling practices of the participants.

Attitude Questions	Participants	Frequency, *n* (%)
How would you rate your knowledge of food safety?	Bad	38 (9.7)
	Fair	260 (66.7)
	Good	92 (23.6)
How often do you get information about food safety knowledge and handling practices in elderly?	Never	94 (24.1)
	Seldom	128 (32.8)
	Sometimes	148 (38.0)
	Always	20 (5.1)
How do you acquire food safety knowledge?	Internet, e.g., social media	313 (80.3)
	Newspaper and book	30 (7.7)
	Radio	15 (3.8)
	Professionals	42 (10.7)
How much more effort do you think you are required in learning safe food handling practices in elderly care?	No effort	1 (0.3)
	Little effort	87 (22.3)
	Moderate effort	179 (45.9)
	High effort	123 (31.5)
In Hong Kong, what is the biggest challenge of the caregivers to follow the safe food handling practices when handling elderly food?	Time-consuming	37 (9.5)
	Inadequate food safety knowledge	249 (63.8)
	Inadequate resources and manpower	104 (26.7)

**Table 4:** Participants’ responses on the Food Safety Knowledge Test.

Knowledge Questions	Correct Answer, *n* (%)	Wrong Answer, *n* (%)
Food Temperature Control and Foodborne Diseases		
1. What is the “Temperature Danger Zone” for microorganisms to grow rapidly?	170 (43.6)	220 (56.4)
2. How long can the food be stored under room temperature (around 25 °C)?	127 (32.6)	263 (67.4)
3. What is/are the cause(s) of foodborne disease?	14 (3.6)	376 (96.4)
Personal and Environmental Hygiene		
4. When should the food handling gloves be changed?	250 (64.1)	140 (35.9)
5. Why do we need to use food-grade sanitizers in kitchen to clean the table surface?	374 (95.9)	16 (4.1)
6. When a food handler has wound on the hands, how should he/she do?	264 (67.7)	126 (32.3)
Safe Food Handling		
7. What temperature should the food be reached when reheat to ensure safety?	109 (27.9)	281 (72.1)
8. What kind of elderly should be provided with soft or minced food?	320 (82.1)	70 (17.9)
9. What is HACCP?	53 (13.6)	337 (86.4)

**Table 5:** Association between perceived and actual food safety knowledge level.

	Poor, *n* (%)	Fair, *n* (%)	Good, *n* (%)	χ^2^, *p*-Value
Perceived Food Safety Knowledge Level	38 (9.7%)	260 (66.7%)	92 (23.6%)	77.14, <0.001
Actual Food Safety Knowledge Level	133 (34.1%)	217 (55.6%)	40 (10.3%)	

**Table 6:** Spearman’s correlation coefficients among 11 variables.

		Gender	Age	Marital Status	Educational Level	Occupation	Monthly Income	Proximity to Elderly	Chronic Disease of Caregivers	Duration of Elderly Caregiving	Knowledge	Practice
**Gender**	r	1.000	0.271 **	−0.063	0.204 **	0.097	0.221 **	0.409 **	0.155 **	0.022	0.217 **	0.037
	*p*-value		<0.001	0.211	<0.001	0.057	<0.001	<0.001	0.002	0.662	<0.001	0.468
**Age**	r	0.271 **	1.000	0.137 **	0.077	0.385 **	0.524 **	0.641 **	0.390 **	0.300 **	0.254 **	0.010
	*p*-value	<0.001		0.007	0.127	<0.001	<0.001	<0.001	<0.001	<0.001	<0.001	0.837
**Marital**	r	−0.063	0.137 **	1.000	−0.117 *	0.065	−0.152 **	−0.122 *	0.164 **	0.275 **	−0.039	0.050
**Status**	*p*-value	0.211	0.007		0.020	0.202	0.003	0.016	0.001	<0.001	0.438	0.328
**Educational**	r	0.204 **	0.077	−0.117 *	1.000	−0.336 **	0.447 **	0.313 **	0.005	0.323 **	0.614 **	0.294 **
**level**	*p*-value	<0.001	0.127	0.020		<0.001	<0.001	<0.001	0.928	<0.001	<0.001	<0.001
**Occupation**	r	0.097	0.385 **	0.065	−0.336 **	1.000	0.212 **	0.347 **	0.221 **	−0.102 *	−0.162 **	−0.192 **
	*p*-value	0.057	<0.001	0.202	<0.001		<0.001	<0.001	<0.001	0.045	0.001	<0.001
**Monthly income**	r	0.221 **	0.524 **	−0.152 **	0.447 **	0.212 **	1.000	0.730 **	0.188 **	0.125 *	0.469 **	0.114 *
	*p*-value	<0.001	<0.001	0.003	<0.001	<0.001		<0.001	<0.001	0.013	<0.001	0.025
**Proximity to elderly**	r	0.409 **	0.641 **	−0.122 *	0.313 **	0.347 **	0.730 **	1.000	0.250 **	0.027	0.380 **	0.044
	*p*-value	<0.001	<0.001	0.016	<0.001	<0.001	<0.001		<0.001	0.602	<0.001	0.382
**Chronic Disease of Caregivers**	r	0.155 **	0.390 **	0.164 **	0.005	0.221 **	0.188 **	0.250 **	1.000	0.244 **	0.066	−0.010
	*p*-value	0.002	<0.001	0.001	0.928	<0.001	<0.001	<0.001		<0.001	0.190	0.838
**Duration of elderly caregiving**	r	0.022	0.300 **	0.275 **	0.323 **	−0.102 *	0.125 *	0.027	0.244 **	1.000	0.354 **	0.246 **
	*p*-value	0.662	<0.001	<0.001	<0.001	0.045	0.013	0.602	<0.001		<0.001	<0.001
**Knowledge**	r	0.217 **	0.254 **	−0.039	0.614 **	−0.162 **	0.469 **	0.380 **	0.066	0.354 **	1.000	0.357 **
	*p*-value	<0.001	<0.001	0.438	<0.001	0.001	<0.001	<0.001	0.190	<0.001		<0.001
**Practice**	r	0.037	0.010	0.050	0.294 **	−0.192 **	0.114 *	0.044	−0.010	0.246 **	0.357 **	1.000
	*p*-value	0.468	0.837	0.328	<0.001	<0.001	0.025	0.382	0.838	<0.001	<0.001	

r: Spearman’s correlation coefficient, *p*-value: asymptotic significance (2-tailed). ** Correlation is significant at the 0.01 level (2-tailed). * Correlation is significant at the 0.05 level (2-tailed).

**Table 7:** Correlations between food safety attitudes, knowledge and practices.

		Chronic Disease of Elderly	Dependency Level	Frequency of symptoms	Perceived Knowledge	Learning Frequency	Effort Required	Knowledge	Practice
**Chronic Disease of Elderly**	r	1.000	−0.252 **	−0.122 *	0.048	−0.013	−0.113 *	0.011	−0.023
	*p*-value		<0.001	0.016	0.344	0.803	0.026	0.826	0.657
**Dependency Level**	r	−0.252 **	1.000	0.128 *	−0.257 **	0.037	0.299 **	−0.026	0.041
	*p*-value	<0.001		0.011	<0.001	0.472	<0.001	0.606	0.415
**Frequency of symptoms**	r	−0.122 *	0.128 *	1.000	−0.203 **	0.066	0.106 *	−0.128 *	−0.122
	*p*-value	0.016	0.011		<0.001	0.195	0.037	0.011	<0.001
**Perceived Knowledge**	r	0.048	−0.257 **	−0.203 **	1.000	0.180 **	−0.344 **	0.464 **	0.204 **
	*p*-value	0.344	<0.001	<0.001		<0.001	<0.001	<0.001	<0.001
**Learning Frequency**	r	−0.013	0.037	0.066	0.180 **	1.000	0.007	0.244 **	0.115 *
	*p*-value	0.803	0.472	0.195	<0.001		0.892	<0.001	0.023
**Effort Required**	r	−0.113 *	0.299 **	.106 *	−0.344 **	0.007	1.000	−0.127 *	0.084
	*p*-value	0.026	<0.001	0.037	<0.001	0.892		0.012	0.097
**Knowledge**	r	0.011	−0.026	−0.128 *	0.464 **	0.244 **	−0.127 *	1.000	0.357 **
	*p*-value	0.826	0.606	0.011	<0.001	<0.001	<0.001		<0.001
**Practice**	r	−0.023	0.041	−0.062	0.204 **	0.115 *	0.084	0.357 **	1.000
	*p*-value	0.657	0.415	0.222	<0.001	0.023	0.097	<0.001	

r: Spearman’s correlation coefficient, *p*-value: asymptotic significance (2-tailed). ** Correlation is significant at the 0.01 level (2-tailed). * Correlation is significant at the 0.05 level (2-tailed).

**Table 8:** Participants’ responses to the food safety handling practices for elderly care.

Questions	Proper Practice n, (%)	Improper Practice n, (%)
Will you check the expiry date when purchasing and handling food?	380 (97.4%)	10 (2.6%)
Will you store the raw food and cooked food separately?	385 (98.7%)	5 (1.3%)
Will you wash your hands thoroughly before handling food?	380 (97.4%)	10 (2.6%)
Will you wash the cooking utensils thoroughly before handling food?	347 (88.9%)	43 (11.1%)
Will you check if the food is safe before serving it to the elderly (e.g., temperature and foreign bodies)?	363 (93.1%)	27 (6.9%)

**Table 9:** Participants’ habits in food handling for elderly care.

Questions	Participants	Frequency, n (%)
Will you store the leftovers?	Yes, store in a sealed container and put in the refrigerator	332 (85.1)
	Yes, store in a sealed container but will not put in the refrigerator	2 (0.5)
	No	56 (14.4)
Do you clean and disinfect the kitchen frequently?	Daily	30 (7.7)
	Weekly	247 (63.5)
	Monthly	86 (22.1)
	Never	26 (6.7)
Will you prepare soft food or minced food for elderly with swallowing difficulties?	Yes	70 (17.9)
	No	39 (10.0)
	I don’t have to take care of elderly with swallowing difficulties	281 (72.1)
Have you applied HACCP when handling food for elderly?	Yes	29 (7.5)
	No	6 (1.5)
	I don’t know what HACCP is	335 (91.0)

## Data Availability

The original contributions presented in the study are included in the article; further inquiries can be directed to the corresponding author.

## Abbreviations

The following abbreviations are used in this manuscript:

FPO	Food poisoning outbreaks
HACCP	Hazard Analysis and Critical Control Points
*d*	Degree of accuracy
*n*	Estimated sample size
*N*	Population size
*P*	Sample portion
*Z*	Confidence interval
χ^2^	Chi-square
r	Spearman’s correlation coefficients
*p*	*p*-value
